# Short‐Term Risk Stratification in Alcohol‐Associated Hepatitis: Small HDL Particles and Inflammation Vulnerability Index Values Improve Model for End‐Stage Liver Disease

**DOI:** 10.1111/acer.70388

**Published:** 2026-08-09

**Authors:** Eduardo Vilar‐Gomez, Margery A. Connelly, Vijay H. Shah, Irina Shalaurova, Jill M. Rafalko, Arun J. Sanyal, Naga P. Chalasani, Samer Gawrieh, TREAT Consortium

**Affiliations:** ^1^ Division of Gastroenterology & Hepatology, Department of Medicine Indiana University School of Medicine Indianapolis Indiana USA; ^2^ Labcorp Morrisville North Carolina USA; ^3^ Division of Gastroenterology and Hepatology Mayo Clinic Rochester Minnesota USA; ^4^ Division of Gastroenterology and Hepatology Virginia Commonwealth University Richmond Virginia USA

**Keywords:** alcohol‐associated hepatitis, inflammation vulnerability index, liver disease, metabolic malnutrition, nuclear magnetic resonance spectroscopy, short‐term mortality

## Abstract

**Background:**

Alcohol‐associated hepatitis (AH) has high short‐term mortality, whereas current prognostic models mainly reflect liver and renal dysfunction. The Metabolic Vulnerability Index (MVX) is a nuclear magnetic resonance‐derived score comprising the Inflammation Vulnerability Index (IVX; small high‐density lipoprotein particles [S‐HDL‐P] and GlycA) and the Metabolic Malnutrition Index (MMX). We evaluated whether MVX, IVX, and IVX components improve 90‐day mortality prediction beyond Model for End‐Stage Liver Disease (MELD) in AH.

**Methods:**

Serum samples from 196 patients with AH, 169 heavy‐drinking controls, and 351 healthy controls were analyzed by nuclear magnetic resonance spectroscopy. The primary outcome was 90‐day AH‐related mortality. Biomarkers were modeled continuously after standardization within the AH cohort; S‐HDL‐P was modeled per 1 SD decrease. Cox models were adjusted for MELD, age, sex, white blood cell count, and baseline antibiotic or corticosteroid use. Model performance was assessed using Akaike information criterion (AIC), likelihood‐ratio testing, and Harrell's *C*‐statistic.

**Results:**

Among patients with AH, 30 AH‐related deaths occurred within 90 days (15.3%). IVX and MVX were highest in AH cases and increased with AH severity, whereas MMX did not consistently differentiate severity. Lower S‐HDL‐P was independently associated with 90‐day mortality in fully adjusted models (HR, 1.84; 95% CI, 1.01–3.35; *p* = 0.045), whereas GlycA was not (HR, 0.86; 95% CI, 0.54–1.37; *p* = 0.527). Adding S‐HDL‐P improved fully adjusted MELD‐based model fit (AIC, 298.97 vs. 301.46; LR *p* = 0.034; *C*‐statistic, 0.741 vs. 0.729). IVX showed supportive improvement in model fit and discrimination (AIC, 299.81; LR *p* = 0.056; *C*‐statistic, 0.749). Continuous MELD interaction analyses supported risk separation for S‐HDL‐P (LR *χ* ^2^ = 6.20; *p* = 0.045) and IVX (LR *χ* ^2^ = 6.64; *p* = 0.036).

**Conclusions:**

Lower S‐HDL‐P appears to be the principal short‐term prognostic component within the IVX domain in AH. S‐HDL‐P and IVX may refine MELD‐based 90‐day risk stratification, pending external validation.

## Graphical abstract

Lower small HDL particles (S‐HDL‐P), the key component of the Inflammation Vulnerability Index, were associated with greater alcohol‐associated hepatitis (AH) severity and higher 90‐day AH‐related mortality. S‐HDL‐P improved Model for End‐Stage Liver Disease (MELD)‐based prognostic model fit, while IVX (inflammation vulnerability index) provided complementary inflammatory‐domain risk information. These findings support S‐HDL‐P and IVX as candidate biomarkers for refining short‐term risk stratification in AH.

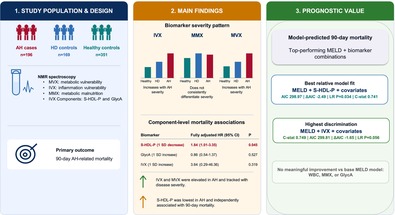

**Abbreviations**
AH: alcohol‐associated hepatitis
ALT: alanine aminotransferase
AST: aspartate aminotransferase
BCAAs: branched chain amino acids
CI: confidence intervals
CVD: cardiovascular disease
HDL: high density lipoprotein
HR: hazard ratio
INR: International Normalized Ratio
IVX: Inflammation Vulnerability Index
MASLD: metabolic dysfunction‐associated steatotic liver disease
MELD: Model for End‐Stage Liver Disease
MMX: Metabolic Malnutrition Index
MVX: Metabolic Vulnerability Index
NMR: nuclear magnetic resonance spectroscopy
S‐HDL‐P: small high‐density lipoprotein particles
TREAT: Translational Research and Evolving Alcoholic Hepatitis Treatment

## 1 Introduction

Alcohol‐associated hepatitis (AH) is a severe form of alcohol‐associated liver disease (ALD) characterized by severe inflammation caused by excessive and prolonged consumption of alcohol (Chaudhry et al. 2023; Crabb et al. 2020). AH is associated with significant morbidity and mortality and is estimated to impact up to 5 million people in the United States alone (Aslam and Kwo 2023). Several factors have been associated with poor outcomes in AH patients, including malnutrition, concurrent chronic hepatitis C, obesity, diabetes mellitus, and metabolic syndrome (Chaudhry et al. 2023; Vilar‐Gomez et al. 2025, 2026). Clinicians commonly use a variety of laboratory tests and prognostic models (such as the Model for End‐stage Liver Disease [MELD], Maddrey Discriminant Function [DF] Score, and Child–Pugh score) to assess the severity of a patient's liver disease, guide treatment decisions, and/or predict a patient's risk of short‐term mortality (i.e., risk for death within 90 days) (Morales‐Arraez et al. 2022; Bataller et al. 2022; Singal et al. 2018).

The Metabolic Vulnerability Index (MVX) is a multi‐analyte, blood‐based prognostic test that has been used to assess 2‐ to 5‐year mortality risk in patients at high risk for cardiovascular disease (CVD) (Otvos et al. 2023). The MVX score is calculated based on the concentrations of six circulating metabolites that are combined into two subscores that reflect the inflammatory and metabolic elements of chronic and end‐stage illness: the Inflammation Vulnerability Index (IVX) and the Metabolic Malnutrition Index (MMX). The IVX sub‐score provides an assessment of systemic inflammation and is calculated using the levels of two biomarkers associated with CVD and mortality: small high‐density lipoprotein particles (S‐HDL‐P) and GlycA. GlycA is a measure of acute‐phase glycosylation patterns, which increase during periods of inflammation. GlycA elevations, coupled with lower levels of S‐HDL‐P, are indicative of inflammation that may be interfering with the protective function of HDL particles, increasing the risk of CVD and mortality (Ajala et al. 2020; Akinkuolie et al. 2014, 2016; Ballout and Remaley 2020; Duprez et al. 2016; Gruppen et al. 2019; Kraus et al. 2022; McGarrah et al. 2017; Otvos et al. 2015). The MMX subscore provides an assessment of metabolic changes associated with malnutrition by measuring the levels of citrate and the branched‐chain amino acids (BCAAs; leucine, isoleucine, and valine). Elevated citrate (which can be a marker of increased energy demand or mitochondrial dysfunction), in the presence of low levels of BCAAs, can indicate protein and caloric deficiencies and is often observed in patients with cancer (cachexia) or end‐stage liver disease (Amjad et al. 2023). When combined, the IVX and MMX scores produce a composite MVX score, ranging from 1 to 100, that is proportional to the patient's relative risk of all‐cause death within 2–5 years, independent of age, sex, and mortality risk factors, such as hypertension, smoking, diabetes, heart disease, and kidney disease (Otvos et al. 2023). As such, MVX may be a useful tool in assessing the risk for mortality due to inflammation or metabolic dysfunction across multiple disease states.

MVX integrates an inflammation domain (IVX: GlycA and small HDL particles) and a malnutrition domain (MMX: branched‐chain amino acids and citrate) and is implemented on a clinical NMR platform as a laboratory‐developed test that clinicians can order. MVX has shown clinical utility in predicting survival vulnerability in patients with heart failure (Conners et al. 2024; Kuku et al. 2025; Kumar et al. 2024), multiple sclerosis (Wicks et al. 2024), and in liver and renal transplant recipients (Fuenzalida et al. 2024; Razzaq et al. 2025) as well as patients with metabolic dysfunction‐associated steatotic liver disease (MASLD) (Lin et al. 2024; Razzaq et al. 2025). The current study evaluated whether MVX, its inflammatory component IVX, its metabolic malnutrition component MMX, and the individual IVX biomarkers S‐HDL‐P and GlycA are associated with AH severity and 90‐day AH‐related mortality. Because MVX and IVX were originally developed in cardiovascular and chronic disease populations, we specifically examined whether the component biomarkers contributed differently in AH. We further evaluated whether S‐HDL‐P and IVX improved prognostic performance beyond MELD and clinical covariates and whether biomarker‐associated risk separation was present across the continuous MELD spectrum.

## 2 Methods

### 2.1 Study Population: AH Cases and Heavy Alcohol Consumption Controls

Subjects were prospectively enrolled in the Translational Research and Evolving Alcoholic Hepatitis Treatment (TREAT) study. Study design, including participants' selection criteria, outcomes definition, assessment, and follow‐up duration, has been published in detail elsewhere (Lourens et al. 2017; Mathur et al. 2021). Briefly, TREAT was a National Institute on Alcohol Abuse and Alcoholism (NIAAA)‐funded multicenter, prospective, observational, case‐controlled study (NCT02172898). All study participants provided written informed consent and were enrolled under an IRB‐approved clinical protocol. The study protocol conformed to the ethical guidelines of the 1975 Declaration of Helsinki. No donor organs were obtained from executed prisoners or other institutionalized persons. Participants enrolled in the AH arm of the study (1) were heavy drinkers, with an average daily ethanol consumption of > 40 g for females and > 60 g for males over a period of at least 6 months and within 6 weeks of study enrollment; (2) had liver disease defined by a serum bilirubin of 3 mg/dL and aspartate aminotransferase (AST) > 50 U/L. Participants with autoimmune liver disease, drug‐induced liver disease, hemochromatosis, Wilson's disease, a history of intravenous drug use, and significant concomitant medical conditions (e.g., uncontrolled congestive heart failure, chronic obstructive pulmonary disease, multiple organ failure) were excluded. Participants enrolled as controls were heavy drinkers (as described above), but without a history, clinical features, or biochemical evidence of liver disease (Mathur et al. 2021; Lourens et al. 2017).

Serum samples, stored at < −70°C until the time of testing, were shipped to Labcorp (Morrisville, NC) for nuclear magnetic resonance (NMR) spectroscopy testing on a Vantera Clinical Analyzer (Labcorp, Morrisville, NC) (Matyus et al. 2014). The digitally stored NMR spectra were used to measure citrate, valine, leucine, isoleucine, small HDL‐P, and GlycA as well as to calculate IVX, MMX, and MVX scores as previously described (Otvos et al. 2023; Wicks et al. 2024). Samples from AH cases (*N* = 196) and matched heavy consumption controls (*N* = 169) from the TREAT study with MVX test results were used for the current study.

### 2.2 Healthy Control Groups

To compare IVX, MMX, and MVX scores from the TREAT cohorts to those of healthy (non‐heavy‐drinking) individuals, NMR spectra from serum samples collected for an additional control cohort, matched by age, sex, and race, were compiled and retrospectively analyzed. The “healthy control cohort” comprised serum samples (*N* = 351) from apparently healthy adult volunteers with no history of chronic disease who donated samples to aid in the establishment of reference ranges at the laboratory and were not part of the TREAT consortium. These samples were tested in 2013, and the NMR spectra were digitized and stored. This study was carried out using an IRB‐approved clinical protocol, and all participants signed consent forms. The study was also conducted in accordance with the Code of Ethics of the Declaration of Helsinki. IVX, MMX, and MVX scores were calculated from digitally stored NMR spectra as previously described (Otvos et al. 2023; Wicks et al. 2024).

### 2.3 Study Outcomes Definition

The primary endpoint was time to 90‐day AH‐related mortality, defined as death directly attributable to liver complications (e.g., liver failure, sepsis), calculated from enrollment. For this study, the primary analysis was limited to the 90‐day follow‐up period. Within this timeframe, 30 deaths directly related to complications of AH were reported, with no deaths occurring in the heavy drinker control group. This timeframe represents a standard benchmark in natural history studies and landmark randomized controlled trials, selected to capture the full window of acute mortality risk, including delayed sepsis and multi‐organ failure endpoints (Gawrieh et al. 2024; Thursz et al. 2015). Deaths were adjudicated according to the TREAT protocol; censoring occurred at 90 days, withdrawal, or last contact. Deaths not adjudicated as AH‐related were censored. As a secondary outcome, we assessed disease severity using the MELD score, defining severe AH as MELD > 20 due to its established prognostic validity (Gaurnizo‐Ortiz et al. 2024; Crabb et al. 2016; Morales‐Arraez et al. 2022).

### 2.4 Statistical Analyses

**FIGURE 1:**
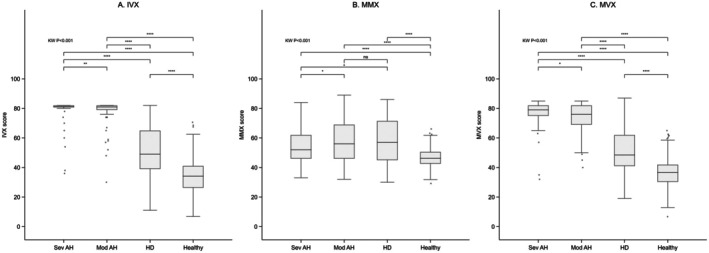
Distribution of IVX, MMX, and MVX scores across alcohol‐associated hepatitis severity groups, heavy drinkers, and healthy controls. Box plots show the distribution of IVX (Panel A), MMX (Panel B), and MVX (Panel C) among individuals with severe alcohol‐associated hepatitis (Sev AH), moderate alcohol‐associated hepatitis (Mod AH), heavy drinkers (HD), and healthy controls. Severe and moderate AH were defined by MELD score > 20 and ≤ 20, respectively. Boxes represent the interquartile range, horizontal lines indicate medians, whiskers represent values within 1.5 times the interquartile range, and circles denote outliers. The global Kruskal–Wallis *p* value is shown within each panel. Pairwise comparisons were performed using Dunn's post hoc test with Holm adjustment; brackets indicate adjusted pairwise comparisons between groups. Significance symbols denote *p < 0.05, **p < 0.01, ***p < 0.001, and ****p < 0.0001; ns indicates not significant. AH, alcohol‐associated hepatitis; HD, heavy drinkers; IVX, Inflammation Vulnerability Index; MELD, Model for End‐Stage Liver Disease; MMX, Metabolic Malnutrition Index; MVX, Metabolic Vulnerability Index.

**FIGURE 2:**
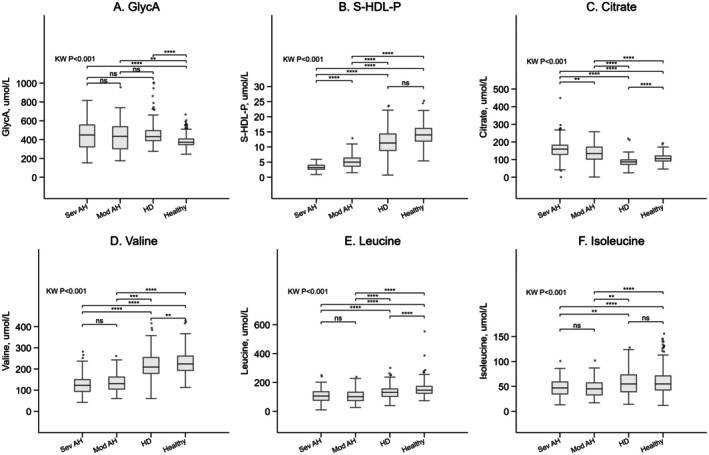
Distribution of IVX and MMX component biomarkers across alcohol‐associated hepatitis severity groups, heavy drinkers, and healthy controls. Box plots show the distribution of IVX components GlycA (Panel A) and small high‐density lipoprotein particles (S‐HDL‐P; Panel B), and MMX components citrate (Panel C), valine (Panel D), leucine (Panel E), and isoleucine (Panel F) among individuals with severe alcohol‐associated hepatitis (Sev AH), moderate alcohol‐associated hepatitis (Mod AH), heavy drinkers (HD), and healthy controls. Severe and moderate AH were defined by MELD score > 20 and ≤ 20, respectively. Boxes represent the interquartile range, horizontal lines indicate medians, whiskers represent values within 1.5 times the interquartile range, and circles denote outliers. The global Kruskal–Wallis *p* value is shown within each panel. Pairwise comparisons were performed using Dunn's post hoc test with Holm adjustment; brackets indicate adjusted pairwise comparisons between groups. Significance symbols denote *p < 0.05, **p < 0.01, ***p < 0.001, and ****p < 0.0001; ns indicates not significant. AH, alcohol‐associated hepatitis; GlycA, nuclear magnetic resonance‐measured glycoprotein acetylation signal; HD, heavy drinkers; IVX, Inflammation Vulnerability Index; MELD, Model for End‐Stage Liver Disease; MMX, Metabolic Malnutrition Index; S‐HDL‐P, small high‐density lipoprotein particles.

Baseline characteristics were summarized as mean ± SD for continuous variables and *n* (%) for categorical variables. Between‐group comparisons were performed using Welch *t*‐tests or Mann–Whitney *U*‐tests for continuous variables and Pearson's *χ* ^2^ or Fisher's exact tests for categorical variables, as appropriate. Multi‐group comparisons (Figures 1 and 2) for biomarker distributions across healthy controls, heavy‐drinking controls, moderate AH, and severe AH were performed using Kruskal–Wallis tests with Dunn's post hoc Holm adjustment. Severe AH was defined by MELD > 20 for descriptive severity comparisons, consistent with established AH severity thresholds (Gaurnizo‐Ortiz et al. 2024; Crabb et al. 2016; Morales‐Arraez et al. 2022). For descriptive analyses across ordered MVX, IVX, and MMX tertiles, *p* values for trend were calculated using Cuzick nonparametric trend tests for continuous severity measures and logistic regression with tertile rank modeled as an ordinal predictor for categorical MELD > 20.

Tertiles were used descriptively to characterize severity gradients across biomarker distributions and to facilitate visualization. MVX, IVX, and MMX tertiles were derived from the combined TREAT cohort and applied to AH cases for descriptive severity analyses. Mortality and prognostic performance analyses modeled biomarkers continuously after standardization within the AH cohort to preserve information, improve generalizability beyond sample‐specific tertile cut points, and reduce instability from sparse events within tertile‐defined strata. Hazard ratios, therefore, represent associations per 1 SD change in the modeled biomarker. S‐HDL‐P was modeled per 1 SD decrease so that higher modeled values reflected lower S‐HDL‐P/higher risk profiles.

Associations between biomarkers and 90‐day AH‐related mortality were evaluated using Cox proportional hazards models. Biomarkers were examined in unadjusted, MELD‐adjusted, and fully adjusted models. The MELD‐adjusted model included the MELD score. The fully adjusted model included the MELD score, age, sex, white blood cell count, baseline antibiotic use, and baseline corticosteroid use. Antibiotic and corticosteroid exposures were treated as baseline concomitant therapies, defined as administration at enrollment or within 48 h. IVX is derived from S‐HDL‐P and GlycA; therefore, S‐HDL‐P and GlycA were also evaluated separately to assess component‐level associations with 90‐day AH‐related mortality.

Prognostic performance was evaluated using nested Cox models among AH participants. The base fully adjusted model included MELD, age, sex, baseline antibiotic use, and baseline corticosteroid use. WBC was evaluated separately as a comparator clinical inflammatory marker and was not included in the base model. S‐HDL‐P, IVX, MVX, MMX, GlycA, and WBC were then added one at a time to the base model. Model fit was assessed using the Akaike information criterion (AIC) and likelihood‐ratio testing, and discrimination was summarized using Harrell's *C*‐statistic. Likelihood‐ratio tests compared each augmented model with the corresponding base MELD + covariate model and were not interpreted as formal tests of differences in Harrell's *C*‐statistic, which was reported as a descriptive measure of discrimination (Burnham and Anderson 2004; Harrell et al. 1984; Harrell 2015). To ensure valid nested model comparisons, all models within each comparison set were estimated using identical complete‐case samples.

To evaluate whether biomarker‐associated risk separation was present across the full spectrum of baseline disease severity without defining new MELD subgroups, fully adjusted Cox models were fitted including continuous MELD, each biomarker, and a continuous MELD‐by‐biomarker interaction term. Global 2‐df likelihood‐ratio tests evaluated the joint contribution of the biomarker main effect and its continuous MELD interaction beyond the fully adjusted MELD‐based model. Model‐predicted 90‐day AH‐related mortality probabilities were plotted across baseline MELD values for the 25th, 50th, and 75th percentiles of each standardized model variable. For S‐HDL‐P, percentile profiles were based on the inverse‐coded standardized model variable, such that higher values represented lower S‐HDL‐P concentrations.

Kaplan–Meier curves by biomarker tertiles were generated as descriptive supplemental visualizations only. Log‐rank *p* values compared survival distributions across tertiles. These tertile‐based Kaplan–Meier analyses were not used as the primary mortality or prognostic performance analyses.

The proportional hazards assumption for Cox models was evaluated using Schoenfeld residual plots and formal tests based on Schoenfeld residuals, with no evidence of violation observed. Adjusted hazard ratios and 95% confidence intervals were calculated to summarize the strength and precision of associations. All statistical analyses were performed using Stata MP Version 19.5 (StataCorp, College Station, TX). Two‐sided *p* values ≤ 0.05 were considered statistically significant.

## 3 Results

### 3.1 Baseline Characteristics of the TREAT Population

A total of 365 participants were included from the TREAT study, comprising 196 patients with AH and 169 heavy‐drinking controls without evidence of AH. Although participants in the parent TREAT study were planned for follow‐up for up to 1 year, the present analysis was restricted a priori to 90‐day AH‐related mortality, consistent with the short‐term prognostic focus of this study. Among patients with AH, the median available follow‐up duration was 259 days (IQR, 161–270). During the full follow‐up period, 47 deaths occurred among AH participants, including 36 deaths adjudicated as directly or indirectly related to AH. Thirty AH‐related deaths occurred within 90 days of enrollment and contributed to the primary survival analysis. Deaths occurring after 90 days, non–AH‐related deaths, and participants censored because of withdrawal or loss to follow up were not counted as primary endpoint events. Among AH participants, 36 withdrew consent or were lost to follow up; in the heavy‐drinking control group, two deaths occurred during follow‐up, and 82 participants were lost to follow up or withdrew consent.

The mean age of the overall TREAT population was 45.0 ± 11.6 years, with no significant difference between heavy‐drinking controls and patients with AH (44.4 ± 12.4 vs. 45.5 ± 10.9 years; *p* = 0.367). Most participants were male (62.2%) and White (84.9%), with no significant differences by AH status for sex (*p* = 0.341) or race (*p* = 0.319) (Table 1).

**TABLE 1:** Baseline characteristics of heavy‐drinking controls and individuals with alcohol‐associated hepatitis in the TREAT cohort.

Characteristic	Heavy‐drinking controls (*N* = 169)	AH cases (*N* = 196)	All participants (*N* = 365)	*p*
Age, years	44.4 ± 12.4	45.5 ± 10.9	45.0 ± 11.6	0.367
Sex				
Female	59 (34.9%)	79 (40.3%)	138 (37.8%)	0.341
Male	110 (65.1%)	117 (59.7%)	227 (62.2%)	
Race				
White	138 (81.7%)	172 (87.8%)	310 (84.9%)	0.319
Black or African American	23 (13.6%)	15 (7.7%)	38 (10.4%)	
Other/unknown	8 (4.7%)	9 (4.6%)	17 (4.7%)	
BMI, kg/m^2^	28.5 ± 6.7	29.1 ± 7.3	28.8 ± 7.0	0.380
MELD score	7.3 ± 2.4	22.5 ± 6.8	15.5 ± 9.2	< 0.001
MELD category				
≤ 20	169 (100.0%)	77 (39.3%)	246 (67.4%)	< 0.001
> 20	0 (0.0%)	119 (60.7%)	119 (32.6%)	
Maddrey DF score	2.8 ± 9.3	43.0 ± 26.7	24.4 ± 28.7	< 0.001
Albumin, g/dL	4.0 ± 0.6	2.8 ± 0.6	3.3 ± 0.8	< 0.001
Total bilirubin, mg/dL	0.6 ± 0.6	14.2 ± 11.0	8.0 ± 10.6	< 0.001
INR	1.0 ± 0.3	1.8 ± 0.5	1.5 ± 0.6	< 0.001
ALT, U/L	26.6 ± 12.4	62.2 ± 63.8	45.8 ± 50.8	< 0.001
AST, U/L	28.5 ± 10.7	136.8 ± 83.5	87.1 ± 82.1	< 0.001
Alkaline phosphatase, U/L	76.3 ± 31.3	188.0 ± 137.1	136.8 ± 117.1	< 0.001
Creatinine, mg/dL	0.9 ± 0.3	1.0 ± 0.9	1.0 ± 0.7	0.056
White blood cell count, 10^9^/L	7.1 ± 2.6	11.6 ± 7.7	9.5 ± 6.4	< 0.001
Baseline antibiotic use	14 (8.3%)	56 (28.6%)	70 (19.2%)	< 0.001
Baseline corticosteroid use	5 (3.0%)	86 (43.9%)	91 (24.9%)	< 0.001
MVX	50.3 ± 14.3	76.4 ± 8.5	64.3 ± 17.4	< 0.001
IVX	51.5 ± 16.6	79.0 ± 7.7	66.2 ± 18.6	< 0.001
MMX	58.5 ± 15.0	55.4 ± 13.0	56.8 ± 14.0	0.038

*Note:* Continuous variables are presented as mean ± SD; categorical variables are presented as *n* (%). Between‐group comparisons were performed for heavy‐drinking controls versus AH cases using Welch *t*‐tests for continuous variables and Pearson's *χ* ^2^ or Fisher's exact tests for categorical variables, as appropriate. Antibiotic and corticosteroid use were defined as baseline concomitant therapies administered at enrollment or within 48 h. Antibiotics included prophylaxis or treatment of suspected/confirmed infection; corticosteroids included systemic corticosteroids given for AH or another inflammatory indication.

Abbreviations: AH, alcohol‐associated hepatitis; ALT, alanine aminotransferase; AST, aspartate aminotransferase; BMI, body mass index; DF, discriminant function; INR, international normalized ratio; IVX, Inflammation Vulnerability Index; MELD, Model for End‐Stage Liver Disease; MMX, Metabolic Malnutrition Index; MVX, Metabolic Vulnerability Index; TREAT, Translational Research and Evolving Alcoholic Hepatitis Treatment.

Compared with heavy‐drinking controls, patients with AH had substantially higher MELD scores (22.5 ± 6.8 vs. 7.3 ± 2.4; *p* < 0.001) and Maddrey discriminant function scores (43.0 ± 26.7 vs. 2.8 ± 9.3; *p* < 0.001). Patients with AH also had higher total bilirubin, INR, ALT, AST, alkaline phosphatase, and WBC and lower albumin than heavy‐drinking controls (all *p* < 0.001) (Table 1).

Baseline antibiotic and corticosteroid use was more frequent among patients with AH than among heavy‐drinking controls (antibiotics: 28.6% vs. 8.3%; corticosteroids: 43.9% vs. 3.0%; both *p* < 0.001). Among AH participants, WBC correlated with MELD score (Pearson *r* = 0.38; *p* < 0.001), but not with MVX (*r* = −0.03; *p* = 0.67), MMX (*r* = 0.04; *p* = 0.57), or IVX (*r* = −0.05; *p* = 0.46).

### 3.2 MMX, IVX, and MVX Scores in TREAT Cohorts Versus Healthy Controls

Mean IVX and MVX scores increased stepwise across groups, with the highest values observed among patients with AH, followed by heavy‐drinking controls and healthy controls. Among AH cases, mean IVX and MVX scores were 79.0 ± 7.7 and 76.4 ± 8.5, respectively, compared with 51.5 ± 16.6 and 50.3 ± 14.3 among heavy‐drinking controls and 33.9 ± 11.5 and 36.4 ± 9.7 among healthy controls (Figure 1; Table S1). IVX and MVX were also higher among participants with severe AH than among those with moderate AH (Figure 1). In contrast, MMX showed a less consistent pattern: mean MMX was higher in the overall TREAT cohort than in healthy controls, but heavy‐drinking controls had slightly higher MMX scores than AH cases (58.5 ± 15.0 vs. 55.4 ± 13.0), and MMX did not differ meaningfully between moderate and severe AH (Figure 1; Table S1).

Evaluation of the component biomarkers showed divergent patterns within the IVX domain. GlycA was elevated among heavy‐drinking controls and patients with AH compared with healthy controls, but GlycA did not distinguish moderate from severe AH. In contrast, S‐HDL‐P was lower among patients with AH than among control groups and was lowest among participants with severe AH (Figure 2). Thus, although both GlycA and S‐HDL‐P contribute to IVX, only S‐HDL‐P showed clear discrimination by AH severity.

Among MMX components, citrate concentrations were highest among participants with severe AH. Branched‐chain amino acid concentrations were lower among AH cases than among heavy‐drinking and healthy controls, particularly for valine; however, these differences did not translate into a consistent MMX severity gradient across moderate and severe AH (Figure 2).

### 3.3 Descriptive Severity Gradients Across MVX, IVX, and MMX Tertiles Among AH Participants

In descriptive tertile analyses among AH participants, MVX and IVX showed ordered associations with AH severity, whereas MMX did not. Across MVX tertiles, mean MELD increased from 17.7 ± 5.5 in the lowest tertile to 23.2 ± 5.9 in the highest tertile (*p* for trend = 0.037), and the proportion with MELD > 20 increased from 14.3% to 67.2% (*p* for trend = 0.006). Maddrey discriminant function scores also increased across MVX tertiles, from 21.7 ± 13.1 to 46.4 ± 24.2 (*p* for trend = 0.005) (Table 2).

**TABLE 2:** Descriptive severity gradients across MVX, IVX, and MMX tertiles among AH participants.

Biomarker/severity feature	T1/lowest	T2/middle	T3/highest	*p* for trend
**MVX**	≤ 57.25	> 57.25 to ≤ 77.17	> 77.17	
*N*	7	73	116	
MELD score	17.7 ± 5.5	22.0 ± 7.8	23.2 ± 5.9	0.037
MELD category				
≤ 20	6 (85.7%)	33 (45.2%)	38 (32.8%)	0.006
> 20	1 (14.3%)	40 (54.8%)	78 (67.2%)	
Maddrey score	21.7 ± 13.1	39.6 ± 30.1	46.4 ± 24.2	0.005
**IVX**	≤ 58.50	> 58.50 to ≤ 80.80	> 80.80	
*N*	8	71	117	
MELD score	19.0 ± 6.0	20.7 ± 6.5	23.9 ± 6.7	< 0.001
MELD category				
≤ 20	6 (75.0%)	35 (49.3%)	36 (30.8%)	0.001
> 20	2 (25.0%)	36 (50.7%)	81 (69.2%)	
Maddrey score	23.7 ± 12.7	35.1 ± 23.9	49.1 ± 27.2	< 0.001
**MMX**	≤ 48.28	> 48.28 to ≤ 62.79	> 62.79	
*N*	67	75	54	
MELD score	22.4 ± 6.5	23.5 ± 6.5	21.4 ± 7.3	0.479
MELD category				
≤ 20	26 (38.8%)	22 (29.3%)	29 (53.7%)	0.131
> 20	41 (61.2%)	53 (70.7%)	25 (46.3%)	
Maddrey score	45.2 ± 26.6	43.5 ± 24.6	39.4 ± 29.5	0.266

*Note:* MVX, IVX, and MMX tertiles were derived from the combined TREAT cohort, including AH cases and heavy‐drinking controls, and were then applied to AH cases for this descriptive analysis. Continuous variables are presented as mean ± SD; categorical variables are presented as *n* (%). *p* values are tests for trend across ordered tertiles; continuous variables were assessed using Cuzick nonparametric trend tests, and categorical MELD > 20 was assessed using logistic regression with tertile rank modeled as an ordinal predictor.

Abbreviations: AH, alcohol‐associated hepatitis; IVX, Inflammation Vulnerability Index; MELD, Model for End‐Stage Liver Disease; MMX, Metabolic Malnutrition Index; MVX, Metabolic Vulnerability Index; TREAT, Translational Research and Evolving Alcoholic Hepatitis Treatment.

A similar, more pronounced severity gradient was observed across IVX tertiles. Mean MELD increased from 19.0 ± 6.0 in the lowest IVX tertile to 23.9 ± 6.7 in the highest tertile (*p* for trend < 0.001), and the proportion with MELD > 20 increased from 25.0% to 69.2% (*p* for trend = 0.001). Maddrey discriminant function scores also increased across IVX tertiles, from 23.7 ± 12.7 to 49.1 ± 27.2 (*p* for trend < 0.001) (Table 2). In contrast, MMX tertiles were not associated with MELD score (P for trend = 0.479), MELD > 20 (*p* for trend = 0.131), or Maddrey discriminant function score (*p* for trend = 0.266). These tertile analyses were used descriptively to characterize biomarker severity gradients; mortality and prognostic performance analyses modeled biomarkers continuously.

### 3.4 Baseline Characteristics by Death Status at 90 Days in the TREAT Population

At 90 days, 30 AH‐related deaths occurred, all among participants with AH. Non‐survivors had higher MELD scores (27.1 ± 5.4 vs. 21.7 ± 6.7; *p* < 0.001), Maddrey discriminant function scores (57.8 ± 23.5 vs. 40.3 ± 26.4; *p* < 0.001), total bilirubin (21.6 ± 11.5 vs. 12.9 ± 10.5 mg/dL; *p* < 0.001), and INR (2.0 ± 0.5 vs. 1.8 ± 0.5; *p* = 0.015) than survivors (Table S2). IVX and MVX, but not MMX, were slightly higher among non‐survivors, although these differences did not reach statistical significance: IVX: 81.3 ± 7.9 vs. 78.5 ± 8.3, *p* = 0.073; MVX: 78.8 ± 4.7 vs. 76.0 ± 9.0, *p* = 0.088; MMX: 55.8 ± 13.4 vs. 55.3 ± 12.9, *p* = 0.861. Among the individual MMX components, citrate concentrations were higher among non‐survivors than survivors (263.5 ± 466.3 vs. 148.8 ± 55.5; *p* = 0.005), whereas valine, leucine, and isoleucine did not differ significantly by 90‐day mortality status (Table S3). WBC did not differ significantly by 90‐day mortality status (*p* = 0.415), supporting its use as a comparator clinical inflammatory marker in subsequent prognostic models.

### 3.5 Continuous Biomarker Associations With 90‐Day AH‐Related Mortality

Because IVX is derived from S‐HDL‐P and GlycA, we evaluated each component separately to determine its relative contribution to 90‐day AH‐related mortality. Lower S‐HDL‐P showed the most consistent component‐level association with mortality. Per 1 SD decrease in S‐HDL‐P, the unadjusted HR was 2.26 (95% CI, 1.32–3.89; *p* = 0.003), the MELD‐adjusted HR was 1.93 (95% CI, 1.08–3.42; *p* = 0.026), and the fully adjusted HR was 1.84 (95% CI, 1.01–3.35; *p* = 0.045) (Table 3). In contrast, GlycA was not independently associated with 90‐day AH‐related mortality in the fully adjusted model (HR, 0.86; 95% CI, 0.54–1.37; *p* = 0.527).

**TABLE 3:** Continuous biomarker associations with 90‐day AH‐related mortality among AH participants.

Biomarker	Scale	Model	HR (95% CI)	*p*
MVX	Per 1 SD increase	Unadjusted	1.61 (0.92–2.81)	0.097
		MELD‐adjusted	1.61 (0.85–3.03)	0.144
		Fully adjusted	1.45 (0.77–2.72)	0.246
IVX	Per 1 SD increase	Unadjusted	13.08 (0.64–267.02)	0.095
		MELD‐adjusted	5.62 (0.30–103.76)	0.246
		Fully adjusted	3.64 (0.29–46.36)	0.319
MMX	Per 1 SD increase	Unadjusted	1.04 (0.73–1.48)	0.844
		MELD‐adjusted	1.05 (0.74–1.50)	0.773
		Fully adjusted	1.11 (0.77–1.59)	0.573
S‐HDL‐P	Per 1 SD decrease	Unadjusted	2.26 (1.32–3.89)	0.003
		MELD‐adjusted	1.93 (1.08–3.42)	0.026
		Fully adjusted	1.84 (1.01–3.35)	0.045
GlycA	Per 1 SD increase	Unadjusted	0.74 (0.50–1.08)	0.115
		MELD‐adjusted	0.81 (0.55–1.18)	0.270
		Fully adjusted	0.86 (0.54–1.37)	0.527

*Note:* Hazard ratios were estimated using Cox proportional hazards models among AH participants. Biomarkers were standardized within the AH cohort and modeled continuously. S‐HDL‐P was modeled per 1 SD decrease so that higher HRs correspond to lower S‐HDL‐P/higher‐risk profiles. The MELD‐adjusted model included the MELD score. The fully adjusted model included MELD score, age, sex, WBC, baseline antibiotic use, and baseline corticosteroid use. IVX is a composite inflammatory vulnerability score derived from S‐HDL‐P and GlycA; therefore, S‐HDL‐P and GlycA were evaluated separately to assess component‐level associations with 90‐day AH‐related mortality.

Abbreviations: AH, alcohol‐associated hepatitis; CI, confidence interval; GlycA, nuclear magnetic resonance‐measured glycoprotein acetylation signal; HR, hazard ratio; IVX, Inflammation Vulnerability Index; MELD, Model for End‐Stage Liver Disease; MMX, Metabolic Malnutrition Index; MVX, Metabolic Vulnerability Index; S‐HDL‐P, small high‐density lipoprotein particles.

When modeled continuously, IVX showed directionally higher risk estimates but wider confidence intervals, and the association was not statistically significant after adjustment. Per 1 SD higher IVX, the unadjusted HR was 13.08 (95% CI, 0.64–267.02; *p* = 0.095), the MELD‐adjusted HR was 5.62 (95% CI, 0.30–103.76; *p* = 0.246), and the fully adjusted HR was 3.64 (95% CI, 0.29–46.36; *p* = 0.319). MVX and MMX were not independently associated with 90‐day AH‐related mortality in fully adjusted continuous models (MVX: HR, 1.45; 95% CI, 0.77–2.72; *p* = 0.246; MMX: HR, 1.11; 95% CI, 0.77–1.59; *p* = 0.573). These findings suggest that S‐HDL‐P is the principal short‐term prognostic component within the IVX domain in AH, whereas GlycA did not show the same direction or strength of association with mortality (Table 3).

Crude Kaplan–Meier curves by biomarker tertiles are presented descriptively in Figure S1. Log‐rank *p* values were 0.015 for S‐HDL‐P, 0.064 for IVX, 0.060 for MVX, and 0.600 for MMX. These tertile‐based analyses were used for visualization only; primary mortality analyses modeled biomarkers continuously.

### 3.6 Prognostic Performance of Biomarker‐Augmented MELD Models

In fully adjusted model performance analyses among AH participants, the base MELD + covariate model had an AIC of 301.46 and a *C*‐statistic of 0.729 (Table 4). Adding WBC did not improve model fit or discrimination (AIC, 303.41; ΔAIC, +1.95; LR *p* = 0.818; *C*‐statistic, 0.730). In contrast, adding S‐HDL‐P improved model fit (AIC, 298.97; ΔAIC, −2.49; LR *p* = 0.034) and modestly increased discrimination (*C*‐statistic, 0.741).

**TABLE 4:** Fully adjusted prognostic performance of biomarker‐augmented MELD models for 90‐day AH‐related mortality among AH participants.

Model	AIC	ΔAIC	LR *p* value	*C*‐statistic
MELD + covariates	301.46	0.00	—	0.729
MELD + WBC + covariates	303.41	+1.95	0.818	0.730
MELD + S‐HDL‐P + covariates	298.97	−2.49	0.034	0.741
MELD + IVX + covariates	299.81	−1.65	0.056	0.749
MELD + MVX + covariates	301.65	+0.19	0.178	0.733
MELD + MMX + covariates	303.21	+1.76	0.621	0.729
MELD + GlycA + covariates	303.00	+1.54	0.498	0.739

*Note:* Covariates included age, sex, baseline antibiotic use, and baseline corticosteroid use. WBC was evaluated as a comparator biomarker/clinical inflammatory marker rather than being included in the base MELD + covariate model. ΔAIC was calculated relative to the base MELD + covariate model; negative values indicate lower AIC and better relative fit. LR *p* values compare each augmented model with the corresponding base MELD + covariate model. *C*‐statistic represents Harrell's concordance statistic.

Abbreviations: AH, alcohol‐associated hepatitis; AIC, Akaike information criterion; GlycA, nuclear magnetic resonance‐measured glycoprotein acetylation signal; IVX, Inflammation Vulnerability Index; LR, likelihood‐ratio; MELD, Model for End‐Stage Liver Disease; MMX, Metabolic Malnutrition Index; MVX, Metabolic Vulnerability Index; S‐HDL‐P, small high‐density lipoprotein particles; WBC, white blood cell count.

Adding IVX also lowered the AIC and yielded the highest *C*‐statistic among the evaluated biomarker‐augmented models, although the likelihood‐ratio test did not meet the conventional 0.05 threshold (AIC, 299.81; ΔAIC, −1.65; LR *p* = 0.056; *C*‐statistic, 0.749). MVX, MMX, and GlycA did not improve model fit relative to the base MELD + covariate model (MVX: ΔAIC, +0.19; LR *p* = 0.178; MMX: ΔAIC, +1.76; LR *p* = 0.621; GlycA: ΔAIC, +1.54; LR *p* = 0.498).

### 3.7 Biomarker‐Associated Risk Separation Across the Continuous MELD Spectrum

We evaluated biomarker‐associated risk separation across continuous MELD values using fully adjusted Cox models that included MELD, each biomarker, and a continuous MELD‐by‐biomarker interaction term. Global 2‐df likelihood‐ratio tests evaluated the joint contribution of each biomarker main effect and its continuous MELD interaction beyond the fully adjusted MELD‐based model. These tests supported the contribution of both S‐HDL‐P (LR *χ* ^2^ = 6.20; *p* = 0.045) and IVX (LR *χ* ^2^ = 6.64; *p* = 0.036).

**FIGURE 3:**
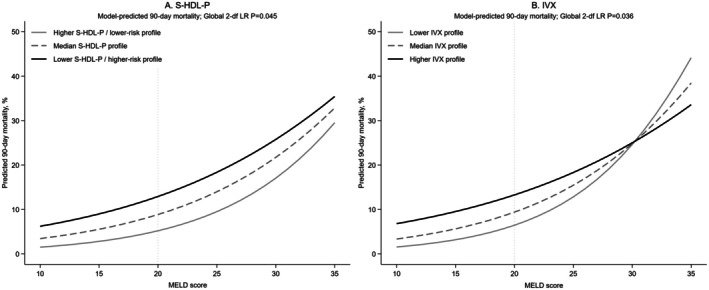
Model‐predicted 90‐day AH‐related mortality according to MELD and biomarker profiles. Model‐predicted 90‐day AH‐related mortality probabilities are shown across baseline MELD values according to S‐HDL‐P and IVX profiles. Curves were derived from fully adjusted Cox models including Meld, each biomarker, and a continuous MELD‐by‐biomarker interaction term, adjusted for age, sex, Wbc, baseline antibiotic use, and baseline corticosteroid use. Profiles represent low, median, and high biomarker values, defined as the 25th, 50th, and 75th percentiles of the continuous biomarker variables among AH participants. These model‐based profiles are distinct from the tertile groups used for descriptive analyses. For S‐HDL‐P, higher values of the modeled biomarker profile correspond to lower S‐HDL‐P concentrations and define the higher risk S‐HDL‐P profile. Global *p* values are from 2‐df likelihood‐ratio tests evaluating the joint contribution of the biomarker main effect and continuous MELD interaction beyond the fully adjusted MELD‐based model. The vertical dotted line at MELD 20 is shown only as a descriptive clinical reference threshold for AH severity and was not used to define the primary prognostic analyses. AH, alcohol‐associated hepatitis; IVX, Inflammation Vulnerability Index; MELD, Model for End‐Stage Liver Disease; S‐HDL‐P, small high‐density lipoprotein particles; WBC, white blood cell count.

Model‐predicted probability curves demonstrated higher 90‐day AH‐related mortality across MELD values for lower S‐HDL‐P/higher risk S‐HDL‐P profiles. For IVX, predicted risk separation was evident across much of the MELD range, although the curves converged and crossed at higher MELD values, where data were relatively sparse (Figure 3).

## 4 Discussion

In this prospective cohort from the TREAT consortium, lower S‐HDL‐P emerged as the most consistent short‐term prognostic biomarker within the IVX inflammatory domain. IVX and MVX were elevated in AH and associated with disease severity, whereas MMX did not consistently differentiate severity. In fully adjusted models, each 1 SD decrease in S‐HDL‐P was associated with higher 90‐day AH‐related mortality (HR, 1.84; 95% CI, 1.01–3.35; *p* = 0.045), while GlycA was not independently associated with mortality (HR, 0.86; 95% CI, 0.54–1.37; *p* = 0.527). Adding S‐HDL‐P to a fully adjusted MELD‐based model improved model fit (ΔAIC, −2.49; LR *p* = 0.034), and IVX showed supportive improvement in model fit and discrimination (ΔAIC, −1.65; LR *p* = 0.056; *C*‐statistic, 0.749). These findings support further evaluation of S‐HDL‐P and IVX as candidate biomarkers for refining MELD‐based short‐term risk stratification in AH.

The prognostic relevance of IVX and S‐HDL‐P may reflect inflammatory and lipoprotein‐related pathways not captured by MELD. MELD primarily reflects liver and renal dysfunction, whereas IVX incorporates two NMR‐derived inflammatory‐domain biomarkers, GlycA and S‐HDL‐P. However, component‐level analyses suggested that these biomarkers contributed differently in AH: lower S‐HDL‐P was associated with greater AH severity and 90‐day AH‐related mortality, whereas GlycA did not independently predict mortality. These findings suggest that S‐HDL‐P may be the principal prognostic component within the IVX domain in this cohort. Although prior studies support biological links between HDL particles, systemic inflammation, and host responses to gut‐derived inflammatory stimuli, the mechanisms linking lower S‐HDL‐P or higher IVX profiles with short‐term AH outcomes were not directly tested in the present study (Connelly et al. 2017; Otvos et al. 2015; Catapano et al. 2014; Chen and Wang 2021; Kim and Seki 2022).

Recent NMR‐based data in patients with cirrhosis further support the relevance of small HDL subclasses in advanced liver disease. Pammer et al. (2025) reported that small and extra‐small HDL particles were depleted in compensated and decompensated cirrhosis, correlated inversely with inflammatory, liver dysfunction, and oxidative stress‐related markers, and independently predicted mortality after adjustment for MELD. These findings suggest that lower S‐HDL‐P may reflect broader HDL remodeling in advanced liver disease, potentially involving inflammatory and oxidative stress‐related pathways; however, these mechanisms remain inferential in our AH cohort and require direct validation.

Because leukocytosis is common in AH and may reflect sterile inflammation, infection, or both, WBC was evaluated as a comparator clinical inflammatory marker. Although WBC correlated with MELD among AH participants, adding WBC to the fully adjusted MELD‐based model did not improve model fit or discrimination (AIC, 303.41; ΔAIC, +1.95; LR *p* = 0.818; *C*‐statistic, 0.730 vs. 0.729 for the base model). In contrast, adding S‐HDL‐P improved model fit and modestly increased discrimination (AIC, 298.97; ΔAIC, −2.49; LR *p* = 0.034; *C*‐statistic, 0.741), while IVX showed supportive improvement in AIC and the highest *C*‐statistic among biomarker‐augmented models (AIC, 299.81; ΔAIC, −1.65; LR *p* = 0.056; *C*‐statistic, 0.749). These findings suggest that S‐HDL‐P and IVX provide prognostic information beyond routine leukocyte count and standard MELD‐based clinical covariates. The weaker performance of MVX may reflect the inclusion of MMX components, which did not show clear short‐term prognostic value.

We further evaluated biomarker‐associated risk separation across continuous MELD values. This approach was chosen to determine whether S‐HDL‐P and IVX add prognostic information across baseline disease severity. Global 2‐df likelihood‐ratio tests supported the joint contribution of S‐HDL‐P and its continuous MELD interaction, as well as IVX and its continuous MELD interaction, beyond the fully adjusted MELD‐based model. These model‐based predicted‐risk analyses showed higher estimated 90‐day AH‐related mortality across MELD values for lower S‐HDL‐P/higher risk S‐HDL‐P profiles. For IVX, risk separation was evident across much of the MELD range, although the curves converged and crossed at higher MELD values where data were relatively sparse. Importantly, these analyses should be interpreted as prognostic visualization across the MELD spectrum rather than evidence for a specific MELD threshold for biomarker use.

Interestingly, the metabolic malnutrition score (MMX) did not predict short‐term mortality. Branched‐chain amino acids were lower in AH than in heavy‐drinking controls, a pattern that may reflect chronic nutritional or metabolic compromise (Dimou et al. 2022; Dasarathy and Merli 2016; Wolfe 2017), but they did not differ meaningfully between survivors and non‐survivors. Thus, in this cohort, BCAA measures did not appear to capture the short‐term mortality signal reflected by S‐HDL‐P or IVX.

In contrast, citrate was higher among non‐survivors, raising the possibility that acute mitochondrial dysfunction or altered intermediary metabolism may contribute to short‐term risk in AH (Li et al. 2024; Mansouri et al. 2018; Hoek et al. 2002). However, because mitochondrial function and TCA‐cycle flux were not directly measured, this interpretation should be considered hypothesis‐generating. The lack of prognostic signal in the composite MMX score may reflect the combination of an acute citrate‐related signal with BCAA measures that are more likely to capture chronic malnutrition or systemic compromise.

These findings extend organ‐centric prognostic models such as MELD by suggesting that systemic inflammatory vulnerability may provide complementary risk information in AH. Although the present study did not directly measure endotoxemia, immune activation pathways, or treatment response, the association of lower S‐HDL‐P and IVX‐related profiles with short‐term risk supports further study of inflammatory‐domain biomarkers as tools for prognostic refinement. Future studies should evaluate whether serial S‐HDL‐P or IVX measurements track clinical trajectory, corticosteroid response, infection risk, or response to novel anti‐inflammatory or microbiome‐directed therapies.

This study has several strengths, including the well‐characterized multicenter TREAT cohort, standardized biospecimen collection, and evaluation of NMR‐derived biomarkers in relation to both AH severity and 90‐day AH‐related mortality. The study also has limitations. The number of 90‐day AH‐related deaths was modest, which limited the precision of some biomarker effect estimates and precluded extensive subgroup modeling. The observational design limits causal inference, and biomarker measurements were obtained at baseline only. Tertile‐based Kaplan–Meier analyses were used descriptively and should not be interpreted as defining clinically validated cut points. The healthy control samples were not part of the TREAT consortium, although they provided useful reference distributions. External validation in independent AH cohorts is required before S‐HDL‐P or IVX can be recommended for clinical risk stratification or treatment decision‐making.

A practical consideration is that MVX and IVX are implemented as NMR‐based laboratory‐developed tests, which may facilitate future translational evaluation if their prognostic value is externally validated in hepatology cohorts. To our knowledge, MVX, IVX, and their component biomarkers have not previously been evaluated for short‐term AH‐related outcomes in this context. Prior work has largely focused on cardiovascular, heart failure, transplant, and metabolic disease populations, underscoring the novelty of applying these indices to AH. However, clinical availability should not be interpreted as evidence of clinical utility in AH until prospective validation and decision‐impact studies are performed.

In conclusion, IVX and MVX were elevated in AH and associated with disease severity, but component‐level analyses showed that lower S‐HDL‐P was the clearest short‐term prognostic biomarker within the IVX domain. S‐HDL‐P improved fully adjusted MELD‐based model performance, and both S‐HDL‐P and IVX contributed to model‐predicted risk separation across the continuous MELD spectrum. GlycA and MMX did not independently predict 90‐day AH‐related mortality. These findings support further validation of S‐HDL‐P and IVX as candidate biomarkers for short‐term prognostic refinement in AH, while emphasizing that mechanisms and clinical utility remain to be established.

## Funding

The Translational Research and Evolving Alcoholic Hepatitis Treatment (TREAT) Consortium was supported by the National Institute on Alcohol Abuse and Alcoholism (NIAAA) (grants: 5U01AA021883‐04, 5U01AA021891‐04, 5U01AA021788‐04, 5U01AA021840‐04, 2U24AA026969‐06). MVX testing was supported by Labcorp.

## Conflicts of Interest

N.P.C. reports no conflicts of interest for this paper. For full disclosure, he reports paid consulting agreements with Madrigal, Zydus, GSK, Altimmune, Eccogene, Akero, Chugai, and Insitro. He has research grants from Boehringer‐Ingelheim, Zydus, and Madrigal. He has equity in Avant Sante, a contract research organization, and Heligenics, a drug discovery start‐up company. S.G. consults for TransMedics and Spruce. He received grants from Zydus. A.J.S. consults for and has other interests in Genfit. He consults for Eli Lilly, Echosens, Abbott, Promed, Satellite Bio, Corcept, Arrowhead, Boston Pharmaceuticals, Variant, Cascade, 89 Bio, AstraZeneca, Alnylam, Regeneron, Boehringer Ingelheim, BMS, Genentech, Gilead, HistoIndex, Janssen, Lipocine, Madrigal, Merck, GSK, Novo Nordisk, PathAI, Pfizer, Poxel, Salix, Myovant, Median Technologies, Sequana, Surrozen, Takeda, Terns, and Zydus. His institution received grants from AstraZeneca, BMS, and Gilead. He owns intellectual property rights with Elsevier and UpToDate. He has other interests in Durect, Tiziana, and Inversago. E.V.‐G. owns intellectual property rights with BMJ Best Practice. V.H.S. has nothing to declare. M.A.C., I.S., and J.M.R. are employees of Labcorp.

## Supporting information


**Figure S1:** Crude Kaplan–Meier estimates of 90‐day AH‐related survival by biomarker tertiles.
**Table S1:** Baseline characteristics in the three populations. Distribution of MVX, MMX, and IVX scores by tertiles in healthy controls, TREAT HD controls and TREAT AH cases.
**Table S2:** Baseline characteristics by death status at 90 days among participants with alcohol‐associated hepatitis (*N* = 196).
**Table S3:** MMX component concentrations according to 90‐day alcohol‐associated hepatitis‐related mortality status.

## Data Availability

The data that support the findings of this study are available from the corresponding author upon reasonable request.
