# Supplementary material for: Short‐Term Risk Stratification in Alcohol‐Associated Hepatitis: Small HDL Particles and Inflammation Vulnerability Index Values Improve Model for End‐Stage Liver Disease
Source: Alcohol Clin Exp Res (Hoboken). 2026 Aug 9;50(8):e70388. doi: 10.1111/acer.70388 (PMC13454315; doi:10.1111/acer.70388)
Supplement: Supplementary file 1 [file ACER-50-0-s001.docx]

**Supplementary Digital Content**

**Short-Term Risk Stratification in Alcohol-Associated Hepatitis: Small HDL Particles and Inflammation Vulnerability Index Values Improve Model for End-Stage Liver Disease**

Eduardo Vilar-Gomez, M.D., Margery A. Connelly, Ph.D., M.B.A., Vijay H. Shah, M.D., Irina Shalaurova, M.D., Ph.D., Jill M. Rafalko, M.S., Arun J. Sanyal, M.D., Naga P. Chalasani, M.D., Samer Gawrieh, M.D., for the TREAT Consortium.

**Table of contents**

|  | Page |
| --- | --- |
| Supplemental Table 1 ------------------------- | 2 |
| Supplemental Table 2 ------------------------- | 3 |
| Supplemental Table 3 ------------------------- | 5 |
| Supplemental Figure 1 ------------------------ | 6 |

**Supplemental Table 1**. Baseline characteristics in the 3 populations. Distribution of MVX, MMX, and IVX scores by tertiles in healthy controls, TREAT HD Controls, and TREAT AH Cases.

|  | Healthy Controls  (N=351) | TREAT  HD Controls  (N=169) | TREAT  AH Cases  (N=196) | TREAT  All  (N=365) |
| --- | --- | --- | --- | --- |
| Age |  |  |  |  |
| Mean years ± SD | 44.3 ± 13.2 | 44.4 ± 12.4 | 45.5 ± 10.9 | 45.0 ± 11.6 |
| Sex |  |  |  |  |
| Female | 131  (37.3%) | 59  (34.9%) | 79  (40.3%) | 138  (37.8%) |
| Male | 220  (62.7%) | 110  (65.1%) | 117  (59.7%) | 227  (62.2%) |
| Race |  |  |  |  |
| Black/AA | 33  (9.4%) | 23  (13.6%) | 15  (7.7%) | 38  (10.4%) |
| White | 294  (83.8%) | 138  (81.7%) | 172  (87.8%) | 310  (84.9%) |
| Other/Unknown | 24  (6.8%) | 8  (4.7%) | 9  (4.6%) | 17  (4.7%) |
| MVX Results |  |  |  |  |
| IVX | 33.9 ± 11.5 | 51.5 ± 16.6 | 79.0 ± 7.7 | 66.2 ± 18.6 |
| ≤58.50 | 341  (97.2%) | 114  (67.5%) | 8  (4.1%) | 122  (33.4%) |
| >58.50 to ≤80.80 | 10  (2.8%) | 51  (30.2%) | 70  (35.7%) | 121  (33.2%) |
| >80.80 | 0  (0.0%) | 4  (2.4%) | 118  (60.2%) | 122  (33.4%) |
| MMX | 46.7 ± 5.9 | 58.5 ± 15.0 | 55.4 ± 13.0 | 56.8 ± 14.0 |
| ≤48.28 | 218  (62.1%) | 55  (32.5%) | 67  (34.2%) | 122  (33.4%) |
| >48.28 to ≤62.79 | 133  (37.9%) | 47  (27.8%) | 75  (38.3%) | 122  (33.4%) |
| >62.79 | 0  (0.0%) | 67  (39.6%) | 54  (27.6%) | 121  (33.2%) |
| MVX | 36.4 ± 9.7 | 50.3 ± 14.3 | 76.4 ± 8.5 | 64.3 ± 17.4 |
| ≤57.25 | 344  (98.0%) | 115  (68.0%) | 7  (3.6%) | 122  (33.4%) |
| >57.25 to ≤77.17 | 7  (2.0%) | 49  (29.0%) | 73  (37.2%) | 122  (33.4%) |
| >77.17 | 0  (0.0%) | 5  (3.0%) | 116  (59.2%) | 121  (33.2%) |

Abbreviations: AA, African American; AH, alcohol-associated hepatitis; ALT, alanine aminotransferase; AST, aspartate aminotransferase; HD, heavy drinkers; MELD, Model for End-Stage Liver Disease; IVX, Inflammation Vulnerability Index; MMX, Metabolic Malnutrition Index; MVX, Metabolic Vulnerability Index; SD, standard deviation; TREAT, Translational Research and Evolving Alcoholic Hepatitis Treatment.

| **Supplemental Table 2.** Baseline characteristics by death status at 90 days among participants with alcohol-associated hepatitis (N=196). | | | | |
| --- | --- | --- | --- | --- |
|  | No | Yes | All | P-value* |
|  | (N=166) | (N=30) | (N=196) |  |
| Age (years) | 45.5 ± 10.9 | 45.7 ± 11.2 | 45.5 ± 10.9 | 0.928 |
| Sex |  |  |  |  |
| Female | 66 (39.8%) | 13 (43.3%) | 79 (40.3%) | 0.713 |
| Male | 100 (60.2%) | 17 (56.7%) | 117 (59.7%) |  |
| Race |  |  |  |  |
| White | 144 (86.7%) | 28 (93.3%) | 172 (87.8%) | 0.616 |
| Black or African American | 13 (7.8%) | 2 (6.7%) | 15 (7.7%) |  |
| Other | 7 (4.2%) | 0 (0.0%) | 7 (3.6%) |  |
| Unknown | 2 (1.2%) | 0 (0.0%) | 2 (1.0%) |  |
| BMI (kg/m2) | 28.9 ± 7.4 | 30.0 ± 6.7 | 29.1 ± 7.3 | 0.446 |
| MELD score | 21.7 ± 6.7 | 27.1 ± 5.4 | 22.5 ± 6.8 | <0.001 |
| MELD cutoffs |  |  |  | <0.001 |
| ≤20 | 75 (45) | 2 (7) | 77 (39) |  |
| >20 | 91 (55) | 28 (93) | 119 (61) |  |
| Maddrey score | 40.3 ± 26.4 | 57.8 ± 23.5 | 43.0 ± 26.7 | <0.001 |
| Albumin (g/dL) | 2.8 ± 0.7 | 2.7 ± 0.5 | 2.8 ± 0.6 | 0.272 |
| Total bilirubin (mg/dl) | 12.9 ± 10.5 | 21.6 ± 11.5 | 14.2 ± 11.0 | <0.001 |
| INR | 1.8 ± 0.5 | 2.0 ± 0.5 | 1.8 ± 0.5 | 0.015 |
| ALT (U/L) | 62.6 ± 66.3 | 59.9 ± 48.7 | 62.2 ± 63.8 | 0.834 |
| AST (U/L) | 137.2 ± 83.2 | 134.6 ± 86.7 | 136.8 ± 83.5 | 0.877 |
| Alkaline phosphatase (U/L) | 188.8 ± 138.3 | 183.5 ± 132.0 | 188.0 ± 137.1 | 0.847 |
| Creatinine (mg/dl) | 1.0 ± 0.9 | 1.2 ± 0.8 | 1.0 ± 0.9 | 0.128 |
| White blood cell (10^9^/L) | 11.4 ± 8.0 | 12.7 ± 5.9 | 11.6 ± 7.7 | 0.415 |
| Antibiotic use † | 46 (28) | 10 (33) | 56 (29) | 0.530 |
| Corticosteroids use † | 68 (41) | 18 (60) | 86 (44) | 0.053 |
| MMX | 55.3 ± 12.9 | 55.8 ± 13.4 | 55.4 ± 13.0 | 0.861 |
| MMX tertiles |  |  |  |  |
| ≤ 48.28 | 57 (34.3%) | 10 (33.3%) | 67 (34.2%) | 0.978 |
| >48.28 to ≤62.79 | 63 (38.0%) | 12 (40.0%) | 75 (38.3%) |  |
| >62.79 | 46 (27.7%) | 8 (26.7%) | 54 (27.6%) |  |
| IVX | 78.5 ± 8.3 | 81.3 ± 7.9 | 79.0 ± 7.7 | 0.073 |
| IVX tertiles |  |  |  |  |
| ≤58.50 | 8 (4.8%) | 0 (0.0%) | 8 (4.1%) | 0.045 |
| >58.50 to ≤80.80 | 64 (38.6%) | 6 (20.0%) | 70 (35.7%) |  |
| >80.80 | 94 (56.6%) | 24 (80.0%) | 118 (60.2%) |  |
| MVX | 76.0 ± 9.0 | 78.8 ± 4.7 | 76.4 ± 8.5 | 0.088 |
| MVX tertiles |  |  |  |  |
| ≤57.25 | 7 (4.2%) | 0 (0.0%) | 7 (3.6%) | 0.085 |
| >57.25 to ≤77.17 | 66 (39.8%) | 7 (23.3%) | 73 (37.2%) |  |
| >77.17 | 93 (56.0%) | 23 (76.7%) | 116 (59.2%) |  |
| Abbreviations: AH, alcohol-associated hepatitis; ALT, alanine aminotransferase; AST, aspartate aminotransferase; BMI, body mass index; INR, International Normalized Ratio; MELD, Model for End-Stage Liver Disease; IVX, Inflammation Vulnerability Index; MMX, Metabolic Malnutrition Index; MVX, Metabolic Vulnerability Index; TREAT, Translational Research and Evolving Alcoholic Hepatitis Treatment.  * t-test or Mann–Whitney across death status for continuous variables, and Pearson's across death status for categorical variables.  † Antibiotic use intended for prophylaxis (spontaneous bacterial peritonitis after variceal bleeding) and treatment of suspected or confirmed infections.  ‡ It represents systemic use of corticosteroids intended for alcoholic hepatitis or any other inflammatory condition. | | | | |

**Supplemental Table 3.** MMX component concentrations according to 90-day alcohol-associated hepatitis -related mortality status.

| **Variable** | **Survivors (N=166)** | **Non-Survivors (N=30)** | **P-value*** |
| --- | --- | --- | --- |
| **MMX Components** |  |  |  |
| Citrate (mean ± SD) | 148.8 ± 55.5 | 263.5 ± 466.3 | **0.005** |
| Valine (mean ± SD) | 129.7 ± 47.5 | 133.9 ± 45.0 | 0.615 |
| Leucine (mean ± SD) | 106.8 ± 45.9 | 100.3 ± 48.6 | 0.472 |
| Isoleucine (mean ± SD) | 47.0 ± 18.1 | 47.6 ± 19.1 | 0.970 |

Abbreviations: MMX, Metabolic Malnutrition Index; SD, standard deviation.

* P values were calculated using the Mann–Whitney U test comparing survivors and non-survivors.

**Supplemental Figure S1.** Crude Kaplan–Meier estimates of 90-day AH-related survival by biomarker tertiles.

Kaplan–Meier curves show unadjusted 90-day AH-related survival among AH participants according to tertiles of S-HDL-P (Panel A), IVX (Panel B), MVX (Panel C), and MMX (Panel D). For S-HDL-P, tertiles were based on the inverse-coded standardized model variable, such that higher values correspond to lower S-HDL-P concentrations and define the higher-risk S-HDL-P profile. Log-rank P values compare survival distributions across tertiles and were 0.015 for S-HDL-P, 0.064 for IVX, 0.060 for MVX, and 0.600 for MMX. Tertile-based Kaplan–Meier analyses were used for descriptive visualization only; primary mortality and prognostic performance analyses modeled biomarkers continuously.

**Abbreviations**: AH, alcohol-associated hepatitis; IVX, Inflammation Vulnerability Index; MMX, Metabolic Malnutrition Index; MVX, Metabolic Vulnerability Index; S-HDL-P, small high-density lipoprotein particles.
